# AGEs in human lens capsule promote the TGFβ2‐mediated EMT of lens epithelial cells: implications for age‐associated fibrosis

**DOI:** 10.1111/acel.12450

**Published:** 2016-02-08

**Authors:** Cibin T. Raghavan, Mareen Smuda, Andrew J. O. Smith, Scott Howell, Dawn G. Smith, Annapurna Singh, Pankaj Gupta, Marcus A. Glomb, Ian Michael Wormstone, Ram H. Nagaraj

**Affiliations:** ^1^ Department of Ophthalmology & Visual SciencesCase Western Reserve University School of MedicineClevelandOHUSA; ^2^ Department of OphthalmologyUniversity of Colorado School of MedicineAuroraCOUSA; ^3^ Institute of ChemistryMartin‐Luther‐University Halle‐WittenbergHalle/SaaleGermany; ^4^ School of Biological SciencesUniversity of East AngliaNorwichUK; ^5^ Visual Sciences Research CenterCase Western Reserve University School of MedicineClevelandOHUSA; ^6^ University Hospitals Eye InstituteClevelandOHUSA

**Keywords:** advanced glycation endproducts, basement membrane, epithelial‐to‐mesenchymal transition, fibrosis, lens epithelial cells, posterior capsular opacification

## Abstract

Proteins in basement membrane (BM) are long‐lived and accumulate chemical modifications during aging; advanced glycation endproduct (AGE) formation is one such modification. The human lens capsule is a BM secreted by lens epithelial cells. In this study, we have investigated the effect of aging and cataracts on the AGE levels in the human lens capsule and determined their role in the epithelial‐to‐mesenchymal transition (EMT) of lens epithelial cells. EMT occurs during posterior capsule opacification (PCO), also known as secondary cataract formation. We found age‐dependent increases in several AGEs and significantly higher levels in cataractous lens capsules than in normal lens capsules measured by LC‐MS/MS. The TGFβ2‐mediated upregulation of the mRNA levels (by qPCR) of EMT‐associated proteins was significantly enhanced in cells cultured on AGE‐modified BM and human lens capsule compared with those on unmodified proteins. Such responses were also observed for TGFβ1. In the human capsular bag model of PCO, the AGE content of the capsule proteins was correlated with the synthesis of TGFβ2‐mediated α‐smooth muscle actin (αSMA). Taken together, our data imply that AGEs in the lens capsule promote the TGFβ2‐mediated fibrosis of lens epithelial cells during PCO and suggest that AGEs in BMs could have a broader role in aging and diabetes‐associated fibrosis.

## Introduction

The human lens capsule is a BM secreted by lens epithelial cells and is composed of interacting networks of laminin and type IV collagen in addition to several heparin sulfate proteoglycans (Danysh & Duncan, 2009). The capsule encloses the lens, sequestering it from other ocular tissue and protecting it from infections. It also provides vital epitopes for the surface receptors of lens cells, which promote lens cell survival, cell migration, and differentiation (Blakely *et al*., 2000; Tholozan *et al*., 2007).

Because BM proteins, including proteins in the lens capsule, are long‐lived, these proteins accumulate post‐synthetic modifications from enzymatic amino acid cross‐linking, lipid peroxidation, and glycation during aging (Sell & Monnier, 1995). Glycation is the reaction of carbonyl compounds, including glucose, ascorbate oxidation products, and methylglyoxal, that forms a variety of structurally diverse stable adducts in proteins; these adducts are collectively known as advanced glycation endproducts or AGEs (Singh *et al*., 2001). Several studies have shown the accumulation of AGEs in the aged BM of the kidneys, lungs, and lens capsule (Bailey *et al*., 1993; Oldfield *et al*., 2001; Song *et al*., 2011).

During cataract surgery, a circular segment of the anterior lens capsule is cut open in a procedure called anterior capsulorhexis (Mohammadpour *et al*., 2012). The lens fiber mass is then emulsified using a probe inserted through this opening and suctioned out in a procedure commonly known as phacoemulsification. The inside lining of the remaining capsule is ‘polished’ to remove anteriorly adhered epithelial cells; next, an artificial intraocular lens is inserted in place of the removed cataract. In most cases, this procedure is safe and initially restores vision. However, in a significant number of patients, epithelial cells that are stubbornly adhered to the remaining anterior capsule move along the capsule to the posterior visual axis behind the intraocular lens and undergo epithelial‐to‐mesenchymal transition (EMT), become fibrotic, and cause wrinkling of the posterior capsule (Wormstone *et al*., 2009). This process is known as posterior capsule opacification (PCO) or secondary cataract formation. PCO impedes vision and requires YAG‐laser treatment to remove the fibrotic mass to clear the visual axis. However, laser treatment could have unintended ill effects, albeit at a low occurrence; these effects can include retinal detachment, macular edema, corneal edema, and displacement of intraocular lens (Billotte & Berdeaux, 2004). Thus, efforts are being made to inhibit PCO development.

Many studies have shown that TGFβ‐mediated signaling through the Smad pathway is essential for lens epithelial cell EMT (Dawes *et al*., 2007; Eldred *et al*., 2011). These studies have proposed that TGFβ is released in response to injury to the lens during cataract surgery and promotes PCO. The role of TGFβ in PCO is supported by many studies in which TGFβ has been shown to induce the expression of α‐smooth muscle actin (αSMA), fibronectin, and collagen type I and III and reduce the expression of vimentin, Snail1/2, and E‐cadherin; these changes are all hallmark features of the EMT (Lee *et al*., 2006; Eldred *et al*., 2011). In addition, aberration in integrin‐mediated cell anchoring has also been implicated in lens epithelial cell EMT (Mamuya *et al*., 2014). Recent studies have also shown a role for aldose reductase, MMP, microRNA, and histone deacetylases in lens epithelial cell EMT (Yadav *et al*., 2009; Wang *et al*., 2013; Korol *et al*., 2014; Xie *et al*., 2014).

Several studies have suggested a role for AGEs in EMT; kidney tubular epithelial cell EMT and fibrosis, which occur in diabetic nephropathy, are mediated through engagement of the receptor for AGEs (RAGE) with AGEs, followed by the synthesis of connective tissue growth factor and can be inhibited by AGE inhibitors (Oldfield *et al*., 2001; Burns *et al*., 2006; Sugimoto *et al*., 2007). The AGE–RAGE interaction also enhances TGFβ1 expression and induces fibrosis of the peritoneal membrane (De Vriese *et al*., 2003). AGE‐modified collagen promotes cardiac fibroblast differentiation into myofibroblasts (Yuen *et al*., 2010), and AGE blockade reduces bleomycin‐induced pulmonary fibrosis in rats (Chen *et al*., 2009). Taken together, these observations support a role for AGEs in fibrotic diseases.

The role of lens capsule AGEs in the EMT that occurs during PCO is not known. Because cataract surgery is common in the elderly, we hypothesized that age‐associated accumulation of AGEs in aged capsules could promote PCO. In this study, we investigated the effect of age on the AGE levels in the lens capsule and then studied the effect of AGE modification in the extracellular matrix on the TGFβ‐mediated EMT in human lens epithelial (HLE) cells.

## Results

### AGE levels increase with age in both anterior and posterior lens capsules

**Figure 1:**
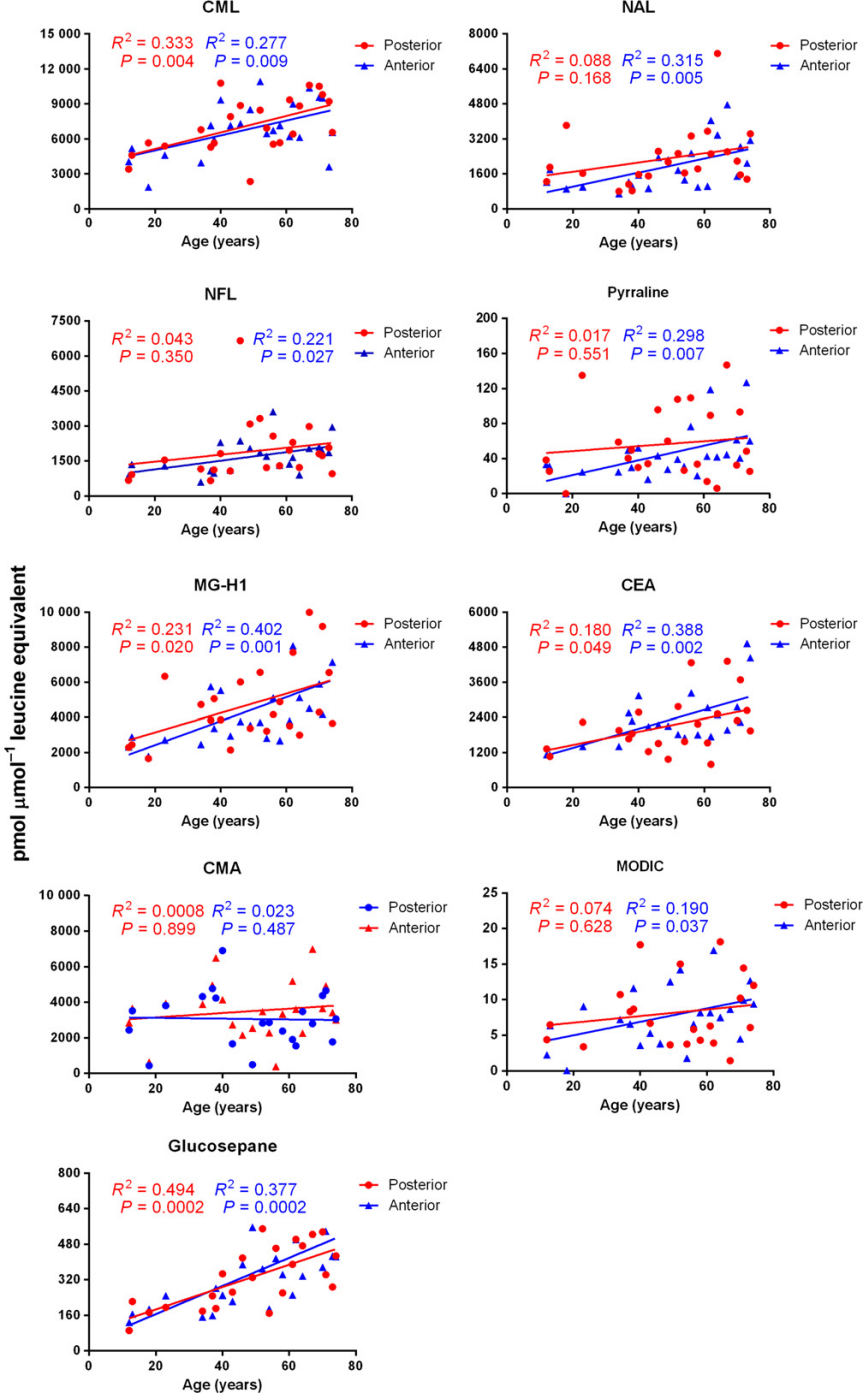
The AGE levels increase in the aging lens capsule. The anterior/posterior portion of the human lens capsules was digested by proteolytic enzymes and analyzed by LC‐MS/MS for AGEs.

We measured 9 AGEs, of which four were exclusively lysine‐derived, three were exclusively arginine‐derived, and two were cross‐linking modifications formed between lysine and arginine residues. All AGE levels are expressed as pmol μmol^−1^ leucine equivalent. In the posterior capsule, AGEs accumulated progressively with age. Among the lysine‐derived AGEs, we measured pyrraline, *N* ^6^‐formyllysine (NFL), *N* ^6^‐acetyllysine (NAL), and CML. CML was the dominant form (Fig. 1). In young lenses (< 30 years), the levels were close to 5000 pmol, and the levels increased to upwards of 10 000 pmol in aged capsules (> 60 years) (Table S2). The second dominant AGE was NAL, which was present at 1000 to 2000 pmol in young capsules and accumulated to levels as high as 4800 pmol in aged capsules. Similarly, NFL and pyrraline were present at ~800 to 1500 pmol, and ~2 to ~30 pmol, respectively, in young capsules and reached ~3400 and ~125 pmol, respectively, in aged capsules. Among the arginine modifications, N^5^‐(5‐hydro‐5‐methyl‐4‐imidazolon‐2‐yl)‐ornithine (MG‐H1) was dominant. This modification was present at approximately 3000 pmol in young capsules and reached as high as 10 000 pmol in aged capsules. The *N* ^6^ *‐*carboxymethylarginine (CMA) levels were more or less steady throughout aging at approximately 3000 pmol. However, the *N* ^6^‐carboxyethylarginine (CEA) levels rose sharply with aging from ~2800 pmol in young capsules to ~6000 pmol in aged capsules. The third category of AGEs was the lysine–arginine cross‐linking structures. We measured glucosepane, which was present at ~125 pmol in young capsules and rose to ~500 pmol in aged capsules. Another lysine–arginine cross‐linking structure, 2‐ammonio‐6‐({2‐[(4‐ammonio‐5‐oxido‐5‐oxopentyl)‐amino]‐4‐methyl‐4,5‐dihydro‐1H‐imidazol‐5‐ylidene}‐amino)‐hexanoate (MODIC), was present in relatively small amounts; the levels were approximately 5 pmol and 15 pmol in young and aged capsules, respectively.

The levels of several AGEs in the anterior capsule were similar to those in the posterior capsule (Fig. 1). For example, the CML, pyrraline, and NAL levels were very similar to the levels in the posterior capsule. However, the levels of NFL were slightly lower and ranged from ~800 pmol in young capsules to ~2000 pmol in aged capsules. Among the arginine modifications, the MG‐H1 and CMA levels were similar to the levels in the posterior capsule, but the CEA levels were slightly lower and ranged from ~1200 pmol in the young capsules to ~4800 pmol in the aged capsules. The levels of the two lysine–arginine cross‐linking structures were very similar to the levels in the posterior capsule. Taken together, these data show that all AGEs accumulated in the capsule with aging and that the rates of accumulation with age were somewhat similar in the anterior and posterior capsules.

We then compared the AGE levels in the posterior and anterior capsules of young (< 30 years) and aged (> 60 years) lenses and found that most AGEs are present at higher levels on both sides of the capsule in aged lenses than in young lenses (Table S2). The greatest difference between the two groups was observed for glucosepane, which was at least 2.5‐fold higher, both in the posterior and anterior sections of the aged capsules than in the young capsules. Most other AGEs increased anywhere from 1.5‐ to 2‐fold in the aged capsules (except for pyrraline and CMA in posterior capsules). These results further confirm the accretion of AGEs in aged capsules.

### AGE levels are higher in cataractous lens capsules than in normal anterior lens capsules

**Figure 2:**
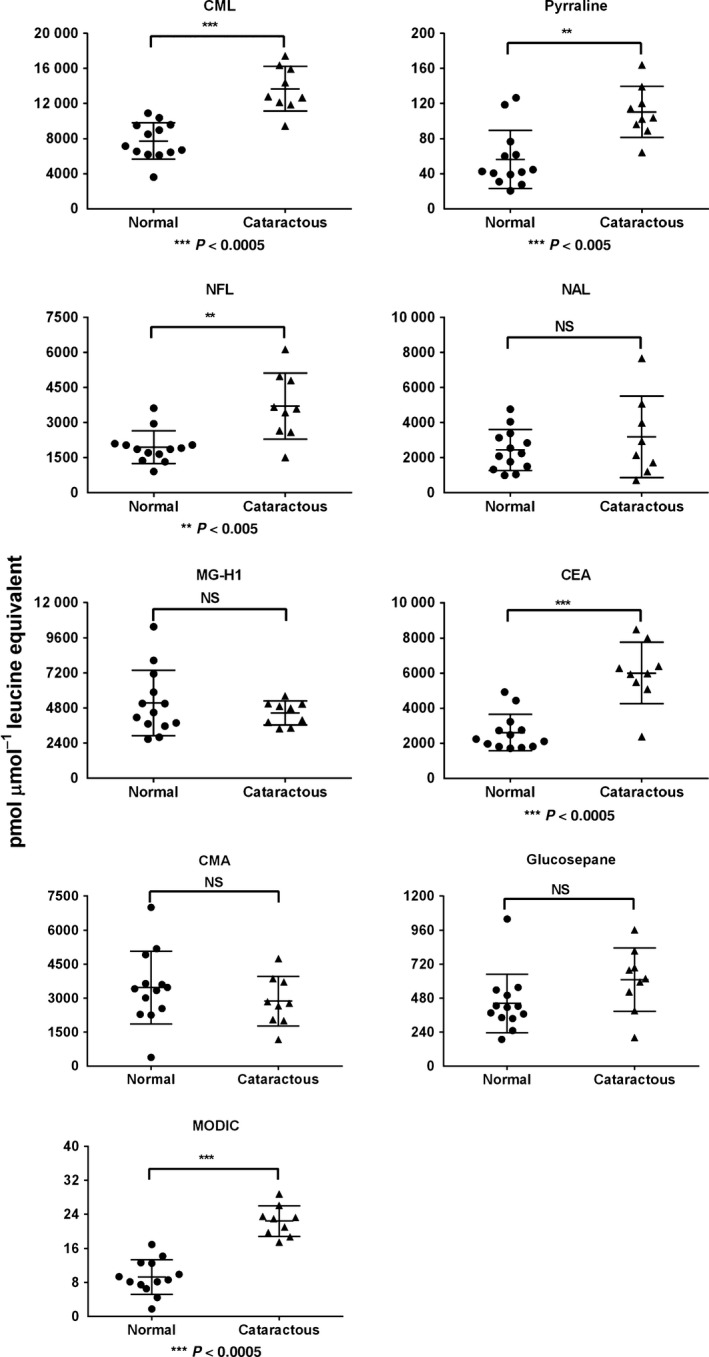
The levels of individual AGEs in cataractous lenses (*n* = 9) are higher than those in normal lenses (*n* = 13). The anterior lens capsules from cataractous and age‐matched normal lenses were analyzed for AGEs by LC‐MS/MS.

We measured the AGE levels in the anterior capsules from cataractous donor lenses (49–75 years), which were obtained during capsulorhexis. The levels of CML ranged between ~3900 and ~10 000 pmol in normal capsules and were significantly (*P* < 0.0005) increased to ~10 000 to 16 200 pmol in cataractous capsules (Fig. 2). A similar pattern of increase was observed for NFL (*P* < 0.005) and pyrraline (*P* < 0.005). The levels of NAL were more or less the same in the cataractous and normal lens capsules. Among the arginine‐derived AGEs, the CEA levels were ~2600 to 5800 pmol in normal capsules and were significantly (*P* < 0.0005) higher and ranged from ~2400 to ~8200 pmol in the cataractous lens capsules. The MG‐H1 and CMA levels were similar between the noncataractous and cataractous lens capsules. The lysine–arginine cross‐linking AGE, MODIC (*P* < 0.0005), was significantly higher in cataractous lens capsules than in normal lens capsules. There was a trend toward an increase, although not significant, in the other lysine–arginine cross‐linking glucosepane in cataractous capsules. The total AGE content was significantly higher across the age range‐ and age‐matched groups in the cataractous lens capsules than in the normal lens capsules (Fig. S1). Taken together, our data indicated that AGEs accumulate in aging lens capsules and that AGEs are formed at higher levels in the capsules of cataractous lenses than in the age‐matched normal lens capsules.

### AGEs in glycated basement membrane extract (BME)

**Figure 3:**
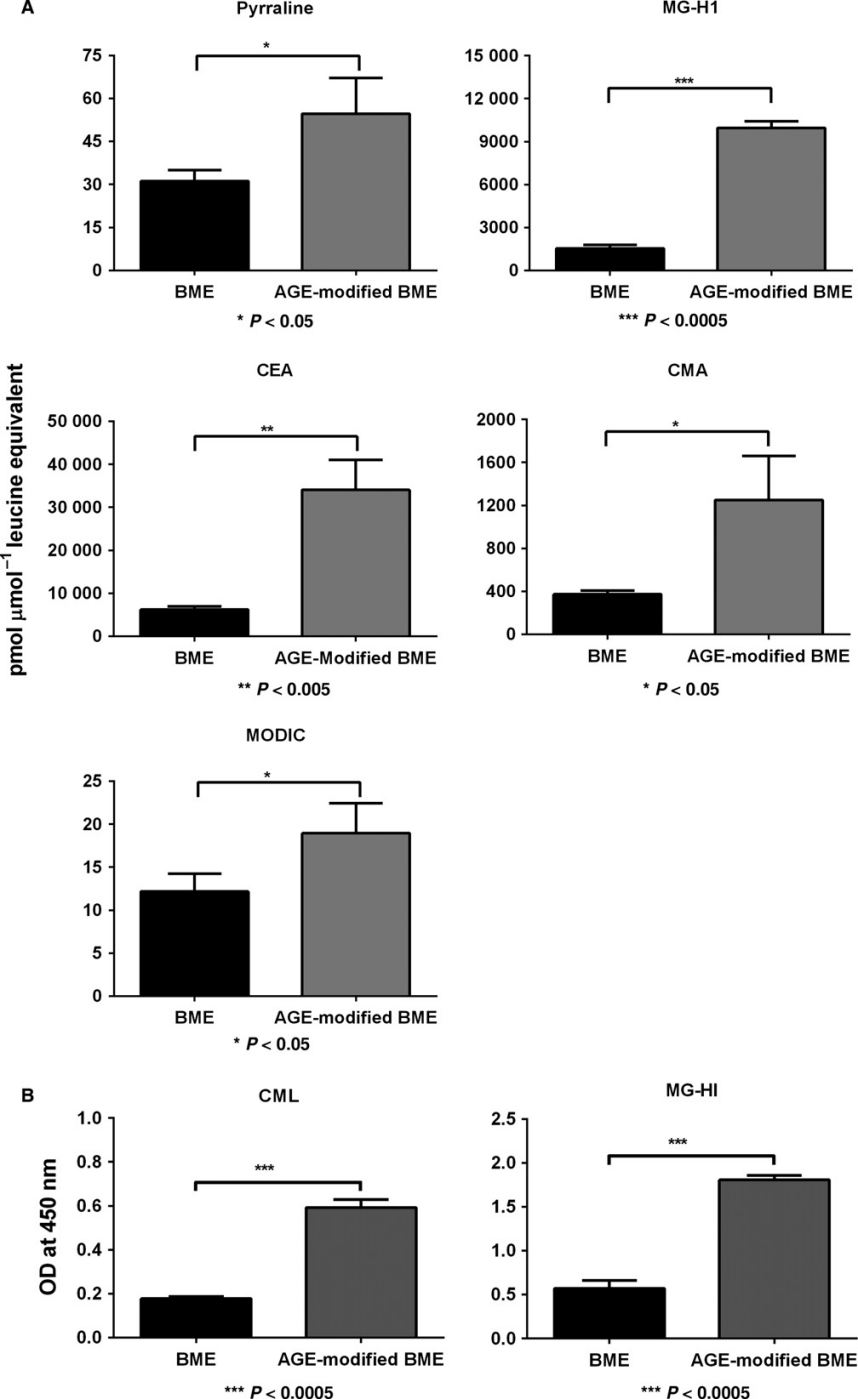
The AGE levels are higher in AGE‐modified BME. To determine whether our glycating mixture was able to generate AGEs in BME, the AGE‐modified or unmodified BME was subjected to LC‐MS/MS analyses for AGEs (A). The bars represent mean ± SD of three independent experiments. To verify whether well‐coated and glycated BME in 96‐well plates had AGE, we measured CML and MG‐H1 using direct ELISAs (B).

Using LC‐MS/MS, we measured 5 AGEs in the AGE‐modified BME. The pyrraline levels significantly (*P* < 0.05) increased from ~30 pmol in unmodified BME to 50 pmol μmol^−1^ leucine equivalents in AGE‐modified BME (Fig. 3). The MG‐H1 levels increased ~fivefold from 2000 pmol in the unmodified BME to 10 000 pmol in AGE‐modified BME (*P* < 0.0005). These levels were comparable to the AGE levels in the human lens capsules; for example, the MG‐H1 levels of ~10 000 pmol in the aged human lens capsules were close to the ~9900 pmol in the AGE‐modified BME. Similarly, the CMA levels were threefold higher in AGE‐modified BME (*P* < 0.05). MODIC was twofold higher in the AGE‐modified BME than in unmodified BME (*P* < 0.05). We could not detect CML in the AGE‐modified BME because of matrix suppression effects. Alternatively, we measured MG‐H1 and CML by ELISAs using specific monoclonal antibodies. Both AGEs were present at significantly higher levels (*P* < 0.0005), which were at least threefold higher in AGE‐modified BME than in unmodified BME (Fig. 3).

### AGE modification of BME enhances the TGFβ2‐induced EMT response in HLE cells

**Figure 4:**
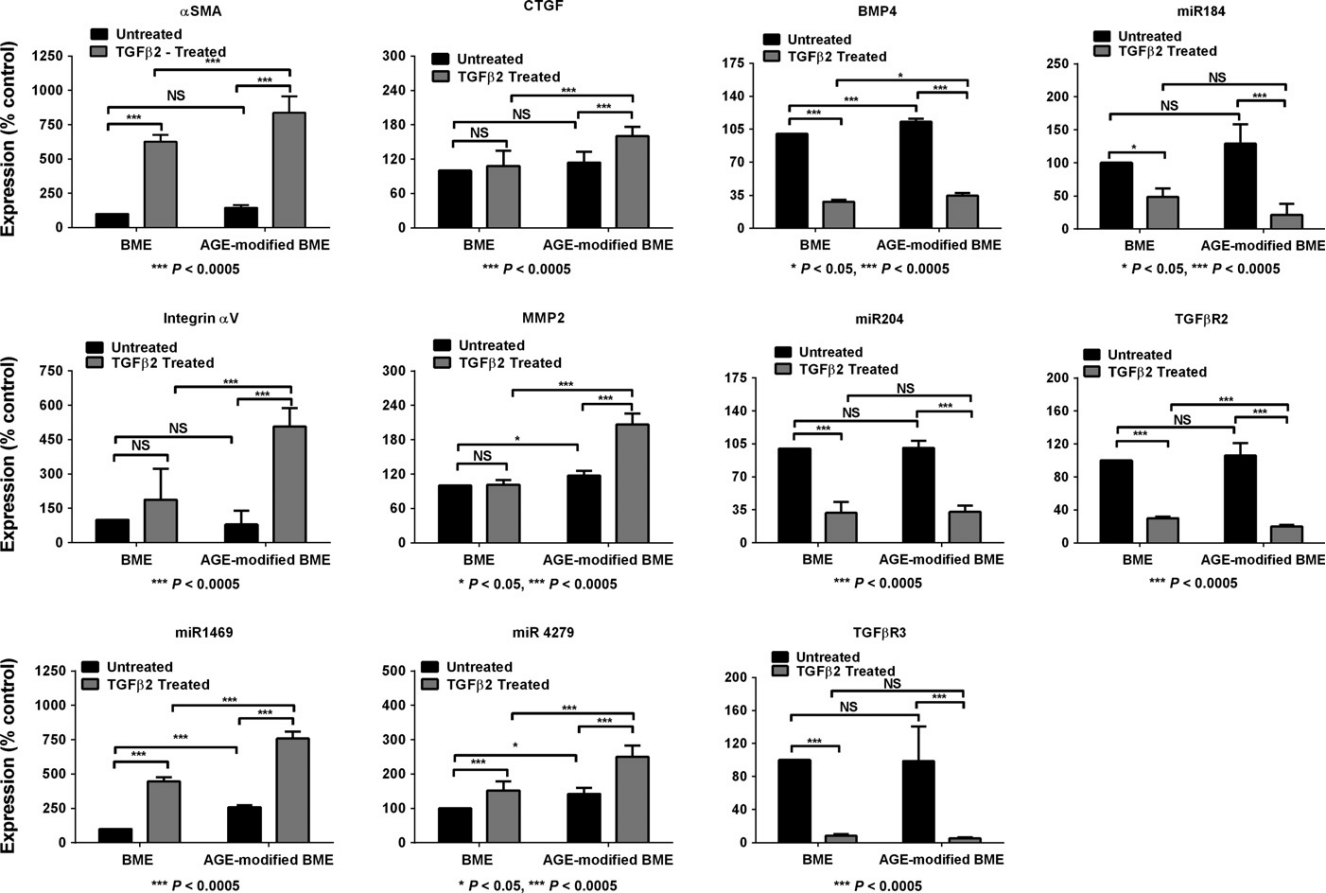
AGE‐modified BME enhances the TGFβ2‐induced EMT marker levels in HLE cells. Lens epithelial cells were cultured on AGE‐modified or unmodified BME and were treated with 10 ng mL^−1^ TGFβ2 for 24 h. The mRNA levels of the EMT markers were quantified by qPCR. The bars represent mean ± SD of four independent experiments.

We next evaluated whether the AGE modification of BME altered the TGFβ2‐induced EMT response in HLE cells. Numerous studies have implicated the TGFβ/Smad signaling pathway in PCO (Saika *et al*., 2004; Wormstone *et al*., 2004). αSMA, MMP2, CTGF, integrin αV, integrin α5, integrin β1, miR4279, and miR1469 have been shown to be upregulated by TGFβ2 during the EMT of HLE cells (Dawes *et al*., 2007; Wang *et al*., 2013; Mamuya *et al*., 2014). Therefore, we measured the mRNA levels of these proteins by qPCR. The AGE‐modified BME (without TGFβ2 treatment) showed significant upregulations in the mRNA levels of MMP2, miR4279, and miR1469, with minor statistically insignificant increases in αSMA, CTGF, and integrin αV. However, the effect of AGEs became more prominent in the presence of TGFβ2. There was a sixfold increase in the mRNA levels of αSMA in the TGFβ2‐treated cells compared with that in the untreated control cells (Fig. 4). This response was eightfold higher in the cells on the AGE‐modified BME compared with cells on the unmodified BME. Similarly, the mRNA levels of CTGF, integrin αV, MMP‐2, miR1469, and miR4279 were significantly higher (*P* < 0.0005) in the cells cultured on AGE‐modified BME than in the cells cultured on unmodified BME. Other known upregulated genes by TGFβ2 in HLE cells, for example, Smad 4 and 7, the integrins α5 and β1, TGFβR1, and fibronectin, were also significantly upregulated in the cells cultured on the AGE‐modified BME compared with the expression on the unmodified BME (Table S4). Our results reveal that the mRNA levels of these proteins were significantly upregulated in HLE cells upon treatment with TGFβ2 and that such upregulation was exaggerated in cells on the AGE‐modified BME.

In contrast to the above results, the mRNA levels of miR204, miR184, TGFβR2, TGFβR3, and BMP4, which have been previously shown to be downregulated by TGFβ2 in HLE cells (Dawes *et al*., 2007; Wang *et al*., 2013), were expectedly downregulated by the TGFβ2 treatment (Fig. 4). However, the mRNA levels of TGFβR2 (*P* < 0.0005) and BMP4 (*P* < 0.05) were further reduced significantly in the cells on the AGE‐modified BME compared with those on the unmodified BME. There were no apparent differences in the mRNA levels of miR204, miR184, and TGFβR2 in the cells on the unmodified or AGE‐modified BME, possibly because these mRNA levels were already highly downregulated by TGFβ2.

We further verified the effect of the AGE‐modified BME on the TGFβ2‐mediated upregulation of αSMA by immunofluorescence. The cells cultured on the unmodified BME and not treated with 10 ng mL^−1^ TGFβ2 showed very little αSMA staining (Fig. S2A). However, the cells in similar conditions but with TGFβ2 treatment showed more αSMA staining. Similar analyses on AGE‐modified BME showed that there was already higher αSMA staining in the cells on the AGE‐modified BME than in the cells on the unmodified BME; upon TGFβ2 treatment, there was a significant increase (*P* < 0.0005) in the αSMA staining. Western blot experiments showed that cells cultured on AGE‐modified BME and TGFβ2‐treated had significantly higher levels of αSMA (*P* < 0.0005) and fibronectin (*P* < 0.0005) than those cultured on unmodified BME (Fig. S2B,C). Moreover, cells cultured on AGE‐modified BME showed an increase in Smad2 phosphorylation after 0.5 h of TGFβ2 treatment. This was accompanied by an increase in pSmad2 translocation to the nucleus (Fig. S3), suggesting the promotion of TGFβ2 signaling by AGEs.

To further verify that AGEs promoted the EMT response of TGFβ2, we used aminoguanidine (AG) to block AGE formation during glycation of BME. While TGFβ2 treatment of cells cultured on AGE‐modified BME resulted in significantly higher mRNA levels of αSMA (*P* < 0.0005) and CTGF (*P* < 0.0005), inclusion of AG during glycation of BME significantly (*P* < 0.0005) reduced that effect (Fig. S4). This confirms that AGEs in BME promote TGFβ2‐mediated EMT of HLE cells.

### AGE modification of the human lens capsule enhances the TGFβ2‐induced EMT response in HLE cells

**Figure 5:**
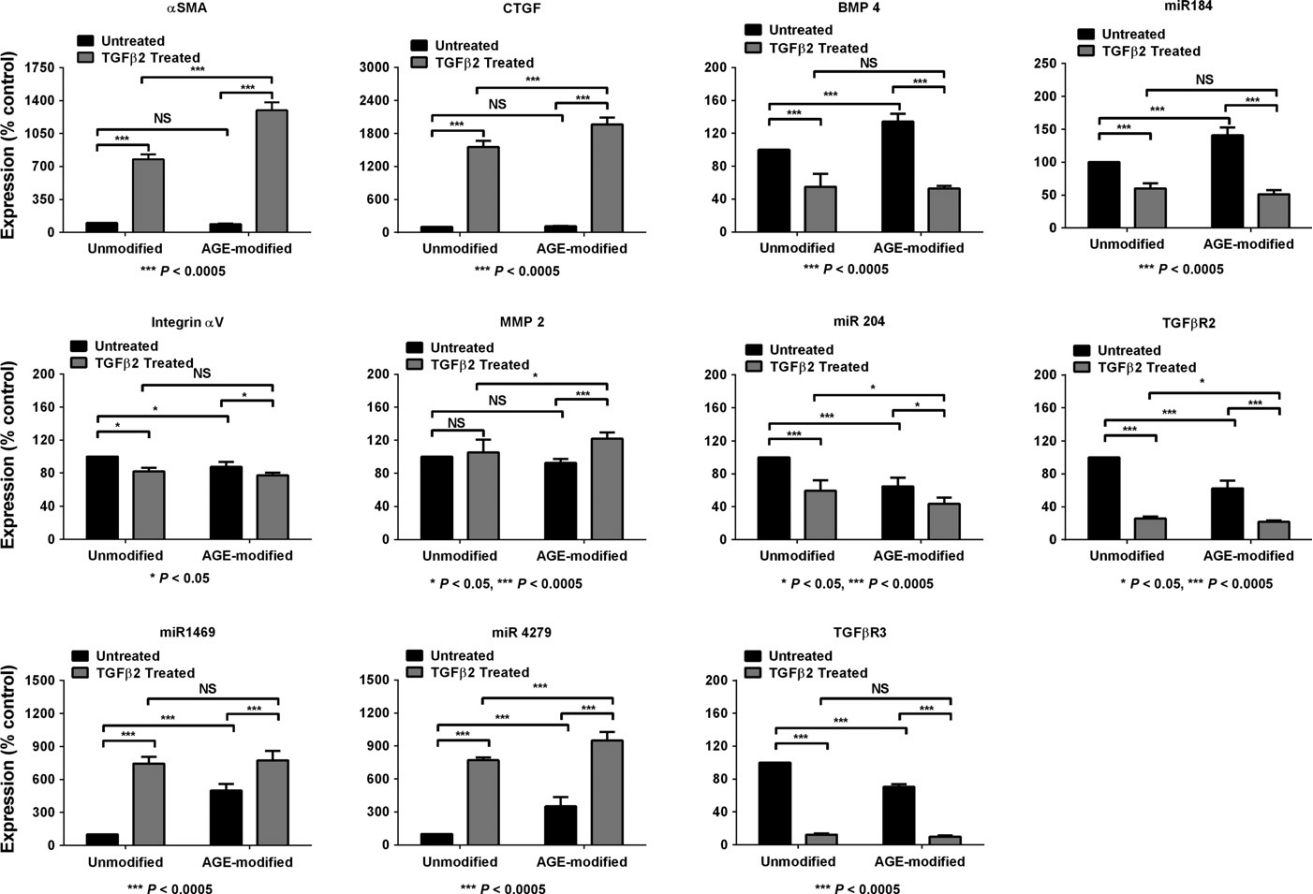
The mRNA levels of EMT markers are elevated in HLE cells cultured on AGE‐modified human lens capsules. Human lens capsules were isolated from young donors, and the adherent epithelial cells were removed. These capsules were then either modified using glycating mixture for 7 days at 37°C or kept as a control (unmodified). HLE cells were cultured on these capsules and treated with 10 ng mL^−1^ TGFβ2 for 24 h, and the mRNA levels of the EMT‐associated protein markers were measured using qPCR. The bars represent mean ± SD of three independent experiments.

We determined the effect of AGE‐modified human lens capsules on HLE cells. One capsule from a pair of capsules from the same donor was AGE‐modified, and the other was similarly incubated in the buffer without the glycating mixture. HLE cells were cultured on these capsule preparations, and when the cells propagated to make a confluent monolayer on the capsule, TGFβ2 was added and the cells were cultured for another 24 h. The EMT response was analyzed using qPCR. In agreement with our results on AGE‐modified BME, the HLE cells cultured on the AGE‐modified capsule showed significant (*P* < 0.0005) increases in the mRNA levels of αSMA, CTGF, and miR4279 over those in cells grown on the unmodified capsule (Fig. 5). Similarly, the MMP2 expression was significantly (*P* < 0.05) elevated in the cells grown on AGE‐modified capsules. Other known upregulated genes by TGFβ2 in HLE cells, for example, fibronectin, integrin α5, and Smad 4 and 7, were also significantly upregulated in the cells cultured on the AGE‐modified capsule compared with the unmodified capsule (Table S5). In contrast, the mRNA levels of miR204 were significantly reduced (*P* < 0.05) in the cells grown on the AGE‐modified capsule compared with those in the cells grown on the unmodified capsule. The mRNA levels of BMP4, miR184, and TGFβR3 were all slightly but statistically insignificantly reduced in the cells grown on the AGE‐modified capsule than in the cells grown on the unmodified capsule. Taken together, these results suggest that the AGE modification of the capsule promotes the EMT response of lens epithelial cells to TGFβ2.

### Relationship between capsule AGEs and the TGFβ2‐mediated EMT response of epithelial cells in the lens capsule

**Figure 6:**
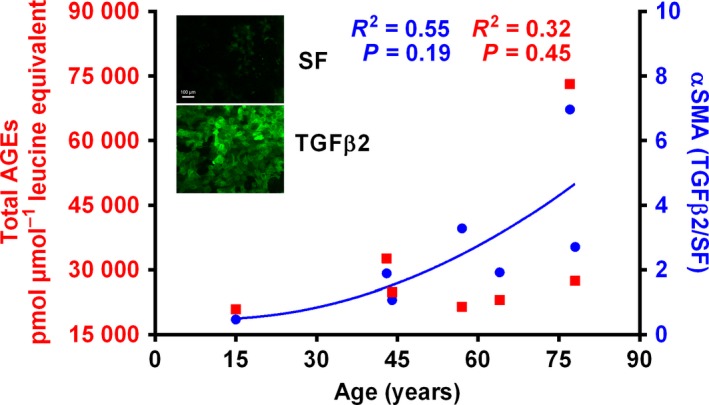
The TGFβ2‐mediated synthesis of αSMA increases with age and is proportional to the AGE content. The human lens capsular bags from 15‐ to 77‐year‐old donors were treated with or without 10 ng mL^−1^ TGFβ2 for 48 h and then maintained for 28 days. The αSMA content was assessed using immunofluorescence. A small portion of the capsule was cut out after the treatment and analyzed by LC‐MS/MS for AGEs. The AGE levels in each capsule are shown in Table S6. The right *Y*‐axis represents the ratio between the fluorescence from αSMA after and before (SF) the TGFβ2 treatment. Inset: Representative images of lens epithelial cells on a 77‐year‐old donor capsule without and with TGFβ2 treatment. Scale bar = 100 μm.

We sought to determine whether there was a relationship between the AGE content of the lens capsule and the EMT response of lens epithelial cells to TGFβ2. For this purpose, we resorted to the human capsular bag model. Capsules of donors of varying age (15–78 years old) were isolated, maintained in culture, and treated with or without 10 ng mL^−1^ TGFβ2 for 2 days and then maintained in SF EMEM until end point (day 28). The AGE levels were measured in small portions of the capsule by LC‐MS/MS. The individual and total AGE levels in each capsule are shown in Table S6. The αSMA levels were measured by immunostaining and analysis using image pro premier software (Media Cybernetics, Warrendale, PA, USA). Interestingly, the αSMA synthesis in response to TGFβ2 showed a weak correlation with the age of the capsule (Fig. 6). More interestingly, the αSMA response to TGFβ2 also correlated with the total AGE content of each capsule. For example, the TGFβ2‐induced αSMA synthesis was highest in the 77‐year‐old capsule, which also had the highest AGE content. Conversely, the TGFβ2‐induced αSMA synthesis was lowest in the 15‐year‐old capsule, which had the lowest levels of AGEs.

We then analyzed the relationship between individual capsule AGEs with the TGFβ2‐mediated αSMA synthesis in HLE cells in capsular bags. Among all the AGEs, NAL, NFL, and MODIC stood out as most significantly related with *P* values of 0.003, 0.009, and 0.005, respectively (Table 1). MG‐H1 and CMA were also significantly related, but not as highly as NAL, NFL, and MODIC.

**Table 1:** Relationship between individual capsule AGEs and αSMA expression by HLE cells in capsular bags

AGEs	*R* ^2^	*P* value
CML	0.560	0.053
NAL	0.839	0.003
NFL	0.768	0.009
Pyrraline	0.557	0.054
MG‐H1	0.758	0.010
CEA	0.384	0.137
CMA	0.696	0.019
MODIC	0.817	0.005

### Matrix protein AGE modification enhances the EMT response to TGFβ1 in HLE cells

Because TGFβ1 is the main isoform of TGFβ in nonocular tissues, we determined whether TGFβ1 had effects similar to those of TGFβ2 on AGE‐modified BME. Our results indicated that the mRNA levels of the major EMT proteins were increased by TGFβ1 and that such an increase was further amplified in the cells cultured on the AGE‐modified BME rather than on the unmodified BME (Fig. S5).

## Discussion

The objectives of this study were to determine whether the age of the lens capsule influences the TGFβ2‐mediated EMT response in lens epithelial cells and whether the AGEs in capsular proteins had any role in this EMT response.

Our observation of the accumulation of structurally different AGEs in the aging capsule suggests that many divergent pathways of glycation are operative in a persistent and chronic manner in human lens capsules. While the accumulation of pyrraline, NAL, NFL, and glucosepane suggests glucose‐mediated glycation (Hayase *et al*., 1989; Glomb & Monnier, 1995), the accumulation of MODIC, CEA, and MG‐H1 suggests glycation by methylglyoxal (Ahmed *et al*., 2002). We would like to note that NAL can also originate from the enzymatic acetylation of lysine residues (Lin *et al*., 1998). Furthermore, the accumulation of CML suggests that in addition to nonoxidative glycation, there is an undercurrent of oxidation in lens capsule modification that (I) triggers fragmentation of the Amadori product of glucose and lysine and (II) triggers formation of glyoxal from glucose and ascorbate oxidation (Glomb & Monnier, 1995).

The higher levels of AGEs in the cataractous lens capsules than in the normal lens capsules are similar to the higher levels of AGEs in cataractous lens proteins (Nagaraj *et al*., 2012) and could be caused by the higher levels of AGE precursors and/or the acceleration of mechanisms for synthesizing AGEs in cataractous lenses. The AGE formation in tissue proteins is enhanced by transition metal ions, oxidative stress, phosphate, and neighboring positively charged amino acids (Chace *et al*., 1991; Breyer *et al*., 2012). Whether any of these factors contributed to the higher level of capsule AGEs in cataractous lenses is not known and needs further investigation.

Our present study had several novelties:

1. Our study showed that lens epithelial cells adhering to young capsules respond feebly to TGFβ2 and consequently synthesize lower levels of αSMA, which is a definitive marker for EMT, than epithelial cells adhering to aged capsules. Children often present the pearl‐type form of PCO and have pronounced Soemmering's ring, which in effect is fiber cell differentiation. It could be that the reduced sensitivity to TGFβ2 in the young lens epithelial cells is linked to their ability to regenerate fiber cells when provided with appropriate stimulus. If a cell goes down the transdifferentiation route, it is following a divergent path to fiber differentiation. AGEs may therefore facilitate EMT and thus promote the fibrotic form of PCO. Therefore, AGE content could be a key determinant in the extent and characteristic PCO features/type an individual patient is likely to present.
2. Our study clearly showed that there is a direct relationship between capsule AGEs and the response to TGFβ2 in HLE cells adhering to the capsule. How AGEs promote the TGFβ2 response is not known, but we can propose a few possibilities. AGEs could interact with RAGE, a receptor of AGEs, and enhance the TGFβ2 response. The interaction between RAGE and AGEs has been implicated in fibrosis in other tissues (Oldfield *et al*., 2001; Song *et al*., 2011). Another possibility is that AGEs in the capsule could affect integrins, which are major conduits between epithelial cells and the lens capsule and promote the TGFβ2‐mediated EMT response. In fact, alterations in integrins, especially αV, have been implicated in PCO (Mamuya *et al*., 2014). It is possible that inhibition of the AGE–RAGE interaction could prevent PCO. These possibilities are currently being investigated in our laboratory.
3. AGEs in general are produced in greater amounts in diabetes because of the underlying hyperglycemia. Several studies, including our own, have provided strong evidence for such a phenomenon (Brownlee *et al*., 1984; Nagaraj *et al*., 2012). Based on our finding in this study that AGEs promote the TGFβ2‐mediated EMT response in HLE cells, it can be speculated that the PCO rate could be higher in individuals with diabetes than in individuals without diabetes. Thus, our study provides a mechanism for the higher rate of PCO in individuals with diabetes.
4. In addition to PCO, fibrosis occurs in many other diseases. In fact, fibrosis in tissues is a major factor for human mortality (McAnulty, 2007). Moreover, the rate of fibrosis increases with age (Calabresi *et al*., 2007). On the basis of the results of this study, we can now explain why this might be the case. It is now well established that the AGE levels in proteins increase with age, especially long‐lived proteins such as those present in basement membranes (Verzijl *et al*., 2000). It is therefore possible that accumulated AGEs promote fibrosis in aged individuals and contribute to disease pathogenesis. Our study could explain why the fibrosis rate is higher in diabetes, for example, in the pathogenesis of diabetic nephropathy (Burns *et al*., 2006). Furthermore, the similar effects of TGFβ1 and TGFβ2 on the AGE enhancement of EMT further suggests that basement membrane AGEs could play a role in TGFβ1‐mediated fibrosis, such as the cardiac and lung fibroses that occur in the diseases of these organs (Yuen *et al*., 2010; Song *et al*., 2011).
5. PCO develops in approximately 20–30% of patients after cataract surgery (Findl *et al*., 2010). It is possible that these patients have higher levels of AGEs in their lens capsules than those who do not develop PCO during the same time period. Future studies on the AGE levels in the anterior capsule obtained during the capsulorhexis procedure of cataract surgery might shed light on this possibility. Furthermore, it might be possible to predict, based on the AGE content in capsulorhexis specimens, the onset of PCO in patients undergoing cataract surgery; this prediction could be used for customized treatments of PCO. Our study showed that the NAL, NFL, and MODIC contents in the capsular proteins are highly related to the TGFβ2‐mediated EMT response in HLE cells. Thus, it might be possible to use these AGEs in capsulorhexis specimens as markers to prospectively predict PCO after cataract surgery.

In summary, our study provided a convincing link between AGEs in the lens capsule and the EMT of HLE cells and could pave the way for future treatment modalities for PCO. This study should also spur research interest in the fibrosis associated with diseases of aging and diabetes.

## Experimental procedures

### Lens capsules

Normal human lens capsules were isolated from frozen donor lenses obtained from the Heartland Lions Eye Bank, Springfield, MO. Anterior capsules from cataractous lenses were collected from the capsulorhexis procedure during cataract surgery at the University Hospitals Eye Institute, Cleveland, OH, with the approval of the Institutional Review Board. Donor eyes for the capsular bag model were obtained from the East Anglian Eye Bank (Norwich, UK). The research was approved by the UK National Ethics Committee (REC 04/Q0102/57) and followed the tenets of the Declaration of Helsinki regarding the use of human material.

### Enzymatic digestion of lens capsules

The capsule was gently peeled away from the lens with the help of tweezers and was cut to separate the anterior and posterior sections with the aid of phase‐contrast microscope (the presence of cells indicated anterior section). The capsules were gently shaken in 0.85% NaCl for 72 h to remove the residual fiber mass and adherent epithelial cells and digested using collagenase and pronase E (Spiro, 1967). Briefly, the specimens were suspended in 0.1 m Tris–acetate buffer, pH 7.4, in the presence of 0.005 m calcium acetate. Collagenase was added on three consecutive days at 0.70%, 0.35%, and 0.10% w/w of the capsule and maintained in a shaker incubator at 37°C. At the end of incubation, undigested material was removed by centrifugation, and the pH was adjusted to 7.8 with 0.5 m Tris. Pronase E at 0.70% (day 1), 0.35% (day 2), and 0.10% w/w (day 3) was used for the subsequent digestion of the capsule. A small crystal of thymol was added at the beginning of the enzymatic digestion to prevent bacterial growth. After digestion, the mixture was passed through a 3‐kDa cutoff filter, vacuum–concentrated, and stored at −86°C until LC‐MS/MS analysis. Before analysis, we measured the amino acid content in the digested material using leucine as the standard, as previously described (Nagaraj *et al*., 1991).

### High performance liquid chromatography–mass spectrometric (LC‐MS/MS) quantitation of AGEs

The HPLC apparatus (Jasco, Groß‐Umstadt, Germany) consisted of a pump (PU‐2080 Plus) with a degasser (LG‐2080‐02) and a quaternary gradient mixer (LG‐2080‐04), a column oven (Jasco Jetstream II) and an autosampler (AS‐2057 Plus). Mass spectrometric detection was conducted on a API 4000 QTrap LC‐MS/MS system (Applied Biosystems/MDS Sciex, Concord, ON, Canada) equipped with a turbo ion spray source using electrospray ionization in positive mode: sprayer capillary voltage of 2.5 kV, nebulizing gas flow of 70 mL min^−1^, heating gas of 80 mL min^−1^ at 650°C, and curtain gas of 30 mL min^−1^. Chromatographic separation of AGEs in the enzyme‐digested capsule samples was performed on a stainless steel column packed with RP‐18 material (KNAUER, 250 × 3.0 mm, Eurospher‐100 C18A, 5 μm, Berlin, Germany) using a flow rate of 0.7 mL min^−1^. The mobile phase used was water (solvent A) and methanol/water (7:3 (v/v), solvent B). To both solvents (A and B), 1.2 mL L^−1^ heptafluorobutyric acid was added. Analysis was performed at a column temperature of 25°C using gradient elution of 2% (0–9.7 min)–10% (16 min)–60% (32 min)–100% (33.5–40.5 min) of solvent B. For mass spectrometric detection, the scheduled multiple reaction monitoring (sMRM) mode was used and consisted of collision‐induced dissociation (CID) of the protonated molecules with compound‐specific orifice potentials and fragment‐specific collision energies (Table 2). The limits of detection (LOD) and quantitation (LOQ) for all monitored compounds are given in Table S1. Quantitation was based on the standard addition method. More precisely, increasing concentrations of authentic reference compounds at factors of 0.5, 1, 2, and 3 times the concentration of the analyte in the sample were added to separate aliquots of the sample. The aliquots were analyzed, and a regression of response vs. concentration was used to determine the concentration of the analyte in the sample. Calibration with this method resolves potential matrix interferences. The AGE LC‐MS/MS method showed coefficients of variation < 5%.

**Table 2:** Mass spectrometric parameters for AGE quantitation

	Retention time	Precursor ion		Product ion 1†			Product ion 2‡			Product ion 3‡		
	Min	*m z* ^−1^	DP/V	*m z* ^−1^	CE/eV	CXP/V	*m z* ^−1^	CE/eV	CXP/V	*m z* ^−1^	CE/eV	CXP/V
CML	3.1	205.1	50.0	130.2	17.0	9.5	84.1	46.0	13.0	56.1	59.0	8.0
NFL	4.7	175.1	40.0	112.1	20.0	13.0	84.1	35.0	7.0	129.1	15.0	13.0
CMA	5.7	233.1	55.0	70.1	27.5	12.0	116.1	23.0	10.0	118.2	22.0	5.5
NAL	6.3	189.2	40.0	126.1	18.0	10.0	84.2	31.0	5.0	143.1	14.0	10.0
CEA	10.7	247.1	51.0	70.2	48.0	11.0	116.2	25.0	10.0	132.1	24.0	10.0
MG‐H1	11.3	229.2	55.0	70.1	43.0	12.0	116.2	20.5	9.0	114.1	22.5	9.0
Glucosepane	23.5/25.4§	429.3	15.0	384.5	41.0	19.0	269.2	55.0	20.0	339.2	55.0	20.0
Pyrraline	26.6	255.2	38.0	175.2	17.0	13.0	237.2	12.0	19.0	148.3	29.0	13.0
MODIC	27.3	357.3	25.0	312.2	35.0	7.0	267.3	45.0	15.0	197.4	45.0	14.0

^†^MRM transition used for quantitation (quantifier), ^‡^MRM transition used for confirmation (qualifier), and ^§^two diastereomeric compounds of glucosepane are present in the capsule.

### AGE modification of basement membrane extract (BME)

Cultrex BME is a soluble form of basement membrane purified from the mouse Engelbreth‐Holm–Swarm tumor (Trevigen, Gaithersburg, MD, USA). The AGE modification of BME was carried out after coating plates with BME (50 μg mL^−1^). The plates were incubated at 37°C with a glycating mixture, consisting of 2 mm ascorbate, 25 mm glucose, and 250 μm methylglyoxal, for 1 week at pH 7.4.

To determine whether the above glycation procedure led to AGE formation, we measured two AGEs. We coated 96‐well cell culture plate wells with 50 μL of BME (50 μg mL^−1^) at 4°C overnight and performed the AGE modification described above. The wells were washed three times with PBST before blocking at room temperature for 2 h with 300 μL of 5% nonfat dry milk in PBST. The wells were washed again three times with PBST and incubated with either a monoclonal antibody for CML or a monoclonal antibody for methylglyoxal‐derived MG‐H1 (both diluted 1:1000 in PBST containing 5% nonfat dry milk, each 50 μL per well) for 1 h at 37°C in a humidified chamber. The 3× PBST‐washed wells were then incubated with 50 μL per well horseradish peroxidase‐conjugated goat anti‐mouse IgG (diluted 1:5000; Promega Corp., Madison, WI, USA) for 1 h at 37°C. The enzyme reaction was assessed by the addition of 100 μL of 3,3,5,5‐tetramethylbenzidine (Sigma‐Aldrich, St Louis, MO) followed by the addition of 50 μL of 2 N sulfuric acid. The chromophore absorbance was measured at 450 nm against a blank well that was coated with BME without AGE modification and processed similarly. In addition, we measured the AGE content (by LC‐MS/MS) in BME suspension (50 μg mL^−1^) glycated with the same glycating mixture as above for 1 week at 37°C.

### Quantitative Real‐Time PCR

HLE cells (~100 000) were seeded on AGE‐modified or unmodified BME in 10‐cm plates in 20% FBS‐MEM and maintained for 2–3 days (to reach 70–75% confluence). The medium was gradually changed to serum‐free medium (10% FBS‐MEM for 24 h and then SF for 24 h) before treatment with 10 ng mL^−1^ TGFβ2 for 24 h. After this treatment, RNA was extracted from the cells using the RNeasy Plus Mini Kit (Qiagen, Valencia, CA, USA). The RNA was reverse transcribed to generate cDNA using the QuantiTect Reverse Transcription Kit (Qiagen). qPCR analysis was performed for the EMT‐associated genes using a LightCycler 96 real‐time PCR system (Roche Life Sciences, Indianapolis, IN, USA). The primers used are listed in Table S3.

### AGE modification of human lens capsules

Human lens capsules from young donors (27–32 years) were gently shaken in 0.85% NaCl for 72 h to remove the residual fiber mass and adherent epithelial cells. The removal of epithelial cells was confirmed using phase‐contrast microscopy. The capsules that were devoid of cells were AGE‐modified for 7 days using the above described glycating mixture at 37°C. The capsules were then pinned onto the culture dish using 0.05‐mm pins so that the posterior side faced up, and the dishes were gently washed with PBS five times before HLE cells were seeded on them. The cells were allowed to attach for 72 h. After this procedure, the cells were deprived of serum and treated with 10 ng mL^−1^ TGFβ2 for 24 h at 37°C. RNA was isolated and cDNA generated as described above. qPCR analysis was performed using the CFX Connect Real‐time PCR system (Bio‐Rad Laboratories, Hercules, CA, USA).

### Human capsular bag model

Following the removal of corneo‐scleral disks for transplantation purposes, donor eyes were obtained from the East Anglian Eye Bank within 48 h of death. A simulated phacoemulsification cataract operation was performed on the donor eyes in a laminar flow hood as previously described (Liu *et al*., 1996). Match‐paired capsular bags were maintained in EMEM supplemented with 50 μg mL^−1^ gentamicin. One preparation within a donor pair was exposed to 10 ng mL^−1^ TGFβ2 (Sigma‐Aldrich) for the first 2 days of culture and then maintained in nonsupplemented EMEM for the remaining culture period. The second preparation was maintained in nonsupplemented EMEM for the entire culture period. The medium was replaced every 2–4 days, and ongoing observations of cell growth were performed using a Nikon phase‐contrast microscope (Nikon, Tokyo, Japan) and a digital camera (Nikon) to capture images. At the experimental end point (day 28), the culture medium was removed from the petri dishes, and the capsular bags were fixed by the addition of 4% formaldehyde (Sigma‐Aldrich).

### Immunohistochemistry

Fixed capsular bag samples, treated as above, were rinsed three times with PBS followed by three washes in a solution of 0.02% w/v BSA and 0.05% v/v IGEPAL (Sigma‐Aldrich) in PBS. The preparations were permeabilized with PBS containing 0.5% v/v Triton X‐100 (Sigma‐Aldrich) for 30 min. Three additional washes with 0.02% w/v BSA and 0.05% v/v IGEPAL in PBS were performed before nonspecific binding sites were blocked with normal goat serum (Sigma‐Aldrich) diluted 1:50 in 1% w/v BSA in PBS for 1 h. Anti‐alpha‐smooth muscle actin (αSMA) mouse monoclonal antibody (Sigma‐Aldrich) was diluted 1:100 with 1% w/v BSA in PBS and applied for 1 h at 37°C. Three washes with 0.02% w/v BSA and 0.05% v/v IGEPAL in PBS were performed, and αSMA was visualized using an Alexa Fluor 488‐conjugated goat anti‐mouse secondary antibody (Invitrogen, Paisley, UK) diluted 1:100 with 1% w/v BSA in PBS for 1 h at 37°C. The preparations were washed a final three times with 0.02% w/v BSA and 0.05% v/v IGEPAL in PBS, floated onto glass microscope slides, and placed in hydromount mounting medium (National Diagnostics, Hull, UK). Images were viewed using a Zeiss epifluorescence microscope and zeiss software (Axiovision). To determine the level of αSMA, image pro premier software (Media Cybernetics) was employed using a method adapted from Wormstone *et al*. (2006). The αSMA level was established by measuring the mean intensity level of all pixels within the fluorescent micrographs.

### Statistics

The data are presented as mean ± SD of the specific number of experiments indicated in the figure legends. The data were analyzed using statview 5.0.1 software (SAS Institute Inc., NC, USA). The statistical significance was evaluated by anova followed by Fisher's protected least significant difference test, and differences were considered significant at *P < *0.05.

## Funding

This work was supported by the National Institutes of Health Grants EY022061, EY023286 (to RHN), and P30EY‐11373 (to Visual Sciences Research Center of Case Western Reserve University), Research to Prevent Blindness, New York (to Case Western Reserve University).

## Conflict of interest

None declared.

## Author contributions

RHN conceived the idea. RHN, IMW, and MAG designed and supervised the work. CTR carried out the qPCR and cell culture experiment. MS performed the LC‐MS/MS experiments. AJOS and IMW performed the capsular bag model. DS carried out cell culture experiments, and SH performed microscopy. AS and PG provided the capsule samples. RHN, IMW, and MAG wrote the manuscript, and all authors reviewed and edited the manuscript.

## Supporting information


**Data S1.** Supporting Experimental Procedures.
**Fig. S1.** Total AGE levels in cataractous and normal lens capsules.
**Fig. S2.** AGE‐BME enhanced the TGFβ2‐mediated expression of EMT markers in HLE cells.
**Fig. S3.** TGFβ2‐mediated Smad2 phosphorylation is increased in HLE cells cultured on AGE‐BME.
**Fig. S4.** Aminoguanidine inhibits the AGE/TGFβ2‐mediated enhancement of EMT response in HLE cells.
**Fig. S5.** AGE‐BME promotes TGFβ1‐mediated EMT response in HLE cells.
**Table S1.** Limit of detection (*LOD)* and limit of quantitation (*LOQ)* of AGEs.
**Table S2.** AGE levels in young vs. aged human lens capsules.
**Table S3.** List of primers used for qPCR.
**Table S4.** Fold change in mRNA levels of EMT markers in HLE cells cultured on unmodified and AGE‐BME and treated with TGFβ2.
**Table S5.** Fold change in mRNA levels of EMT markers in HLE cells cultured on unmodified and AGE‐modified lens capsules and treated with TGFβ2.
**Table S6.** Individual and total AGE levels in capsule specimens from capsular bags.
