# Supplementary material for: AGEs in human lens capsule promote the TGFβ2‐mediated EMT of lens epithelial cells: implications for age‐associated fibrosis
Source: Aging Cell. 2016 Feb 8;15(3):465–76. doi: 10.1111/acel.12450 (PMC4854921; doi:10.1111/acel.12450)
Supplement: Supplementary file 1 — Data S1. Supporting Experimental Procedures. Fig. S1. Total AGE levels in cataractous and normal lens capsules. Fig. S2. AGE‐BME enhanced the TGFβ2‐mediated expression of EMT markers in HLE cells. Fig. S3. TGFβ2‐mediated Smad2 phosphorylation is increased in HLE cells cultured on AGE‐BME. Fig. S4. Aminoguanidine inhibits the AGE/TGFβ2‐mediated enhancement of EMT response in HLE cells. Fig. S5. AGE‐BME promotes TGFβ1‐mediated EMT response in HLE cells. Table S1. Limit of detection (LOD) and limit of quantitation (LOQ) of AGEs. Table S2. AGE levels in young vs. aged human lens capsules. Table S3. List of primers used for qPCR. Table S4. Fold change in mRNA levels of EMT markers in HLE cells cultured on unmodified and AGE‐BME and treated with TGFβ2. Table S5. Fold change in mRNA levels of EMT markers in HLE cells cultured on unmodified and AGE‐modified lens capsules and treated with TGFβ2. Table S6. Individual and total AGE levels in capsule specimens from capsular bags. [file ACEL-15-465-s001.docx]

**Supporting Experimental procedures**

**Isolation and culture of HLE cells.**

HLE cells were isolated from the lenses of a 48-year-old donor with no systemic or eye diseases. Briefly, the lenses were removed from the whole eye globes (received within 40 h of death from Midwest Eye-Banks, Ann Arbor, MI) using a posterior dissection approach. All adhering tissues were removed, and the lenses were gently washed with PBS containing 100 μg of streptomycin and 100 U penicillin per ml. The lenses were then placed in a 60-mm tissue culture plate with 2 ml of 0.05% trypsin/EDTA and minced with a sterile scalpel. The preparation was incubated in a humidified atmosphere at 5% CO_2_ at 37 °C for 10 min with frequent swirling. Next, 5 ml of minimum essential medium (MEM) containing 20% fetal bovine serum (FBS), 2 mM L-glutamine and 50 μg/ml gentamycin was added, and the contents were transferred into a 15-ml conical tube. The plate was washed with 5 ml of PBS, and the contents of the plate were added to the same conical tube. The contents were centrifuged at 1,000 rpm for 10 min, and the tissue pellets were re-suspended in 10 ml of 20% MEM containing 2 mM L-glutamine and 50 μg/ml gentamycin. This mixture was transferred to a 100-mm tissue culture plate and incubated in a tissue culture incubator_._ Then, 1 ml of medium was added every 3 days. After 2 weeks, the medium was changed, and the cell growth was observed. Lens epithelial cells were characterized by staining with a monoclonal antibody for human αB-crystallin (1:100 dilution; Enzo Life Sciences, Farmingdale, NY). Cells between passages 4 and 7 were used in all studies.

# Immunocytochemistry.

# Six-well culture plates were coated with BME and AGE-modified as described above. HLE cells were seeded and cultured on AGE-modified or unmodified BME until the cells were 80% confluent. The cells were deprived of serum for 24 h before TGFβ2 treatment (10 ng/ml) for 24 h. After 24 h of TGFβ2 treatment, the cells were fixed with 4% paraformaldehyde in PBS for 20 min at room temperature, followed by three washes with PBS. The cells were then permeabilized with ice cold 80% methanol in PBS for 15 min at -20 °C. After being blocked with 5% normal goat serum in PBS, the cells were incubated overnight at 4 °C with a monoclonal antibody for αSMA (1:500 dilution, Sigma Aldrich, St Louis, MO) followed by 1 h of incubation at 37 °C with an Oregon Green 488 goat anti-mouse IgG (1:250 dilution, Life Technologies, Carlsbad, CA). The cells were permanently mounted with DAPI/Vectashield for observations of the nuclei.

**Western blotting.**

Whole cell lysates (for αSMA and fibronectin) were prepared using the Mammalian Protein Extraction Reagent (Thermo scientific, Rockford, IL) containing a 1:100 diluted protease and phosphatase inhibitor cocktail (Sigma-Aldrich). Cytosolic and nuclear fractions (for pSmad2) were prepared using the NucBuster protein extraction reagent (Millipore, Billerica, MA) containing a 1:100 diluted protease and phosphatase inhibitor cocktail (Sigma-Aldrich). Proteins (10 to 20 µg) were separated on 8% or 12% SDS-PAGE and transferred to a nitrocellulose membrane. The membranes were incubated at 4 °C overnight with primary antibodies against the following proteins: αSMA (1:5000 dilution, Sigma-Aldrich), Fibronectin (1:200 dilution**,** Santa Cruz Biotechnology, Dallas, TX), phosphorylated and total Smad2 (S245/250/255) and β-Actin (1:1000 dilution, Cell Signaling, Danvers, MA). Appropriate HRP-conjugated secondary antibodies (Cell Signaling) were used. The protein bands were detected using the SuperSignal West Pico or Femto Kit (Pierce Chemicals, IL).

**AGE modification of BME in the presence of aminoguanidine.**

The AGE modification of BME was carried out as mentioned in experimental procedures. After coating plates with BME (50 μg/ml), the plates were incubated at 37 °C with the glycating mixture (2 mM ascorbate, 25 mM glucose and 250 μM methylglyoxal) and 1 mM aminoguanidine, for one week.

**Supporting Results**

**LC-MS/MS in-source oxidation control experiments**

It was possible that some amino acid derivatives could generate oxidation artifacts at the source of the LC-MS/MS system during electrospray ionization ([Thornalley & Rabbani 2014](#_ENREF_3)). To exclude overestimation of *N*^6^-carboxymethyllysine (CML) resulting from oxidation of *N*^ε^-fructose lysine (FL), it was essential that CML and FL were chromatographically separated. In the method used herein the retention time of CML and FL were 3.1 and 4.1 min, respectively. Additionally, a few samples were reduced chemically to exclude FL as a CML precursor as follows. Three pools of capsules, each containing three individual specimen (all from 65 year donors) were digested with collagenase and pronase E as described in methods section. Samples were lyophilized after MWCO filtration (3,000 Da) and residues were dissolved in de-ionized water. One aliquot was reduced with a solution of NaBH_4_ (20 mg/ml in 0.01 N NaOH; equimolar amounts of NaBH_4_ to leucine equivalents were used) and another was treated with the same volume of 0.01 N NaOH without the reducing agent. Samples were kept at RT for 1 h. Subsequently 3 N HCl was added and solutions were vacuum concentrated. Residues were dissolved in de-ionized water and diluted to appropriate concentrations for LC-MS/MS measurements. The CML values for reduced compared to non-reduced samples differed only by +/- 5 %. Thus, we exclude the possibility that CML was artificially generated from FL during LC-MS/MS.

# Supporting Figure legends

**FIGURE S1.** Total AGE levels are higher across the age range (49 to 75 years) in cataractous lens capsules (n = 9) than in normal lens capsules (n = 13). The aggregate AGE levels were higher in the anterior capsules of cataractous lenses than in normal lenses across the age range (A) studied. The aggregate AGE levels were significantly higher in the cataractous capsule group than in the normal capsule group (B). The bars represent the mean ± SD of the AGE levels.

**FIGURE S2.** The expression of αSMA is increased in HLE cells cultured on AGE-modified BME. Lens epithelial cells were cultured on unmodified and AGE-modified BME. αSMA was detected using a monoclonal antibody against αSMA and Oregon green 488-goat anti-mouse IgG. The images shown are representative ones from three independent experiments (A). The fluorescence intensity was measured using MetaMorph software (Molecular Devices, Sunnyvale, CA), and the intensity plot is shown on the right. Scale bar = 50 μm. Western blotting analysis was carried out for αSMA (B) and fibronectin (C) with whole cell lysate (after 48h TGFβ2 treatment) using the respective primary antibodies as mentioned above. Densitometric analyses from three independent assays (mean ± SD) are shown in the bar graphs.

**FIGURE S3.** Smad2 phosphorylation is increased in HLE cultured on AGE-modified BME and treated with TGFβ2. Lens epithelial cells were cultured on unmodified and AGE-modified BME, treated with TGFβ2 for various time periods. Cytosolic and nuclear fractions of TGF-β2-treated cells were prepared as mentioned in supporting experimental procedures. The effect of AGE-modified BME on Smad2 phosphorylation is shown in A and B. Densitometric analyses from 4 independent assays (mean ± SD) are shown in the bar graphs.

**FIGURE S4.** Aminoguanidine inhibits the TGFβ2-mediated EMT response in HLE cells grown on AGE-modified BME. AGE-modification was carried out in the presence of 1 mM aminoguanidine. HLE cells were cultured with 10 ng/ml TGFβ2 for 24 h. The mRNA levels of the EMT-associated proteins – αSMA and CTGF were quantified using qPCR. The bars represent the mean ± SD of three independent experiments.

**FIGURE S5.** The response to TGFβ1 is similar to the response to TFGβ2 in HLE cells grown on AGE-modified BME. HLE cells were cultured on AGE-modified or unmodified BME and treated with 10 ng/ml TGFβ1 for 24 h. The mRNA levels of the EMT-associated proteins were quantified using qPCR. The bars represent the mean ± SD of three independent experiments.

**

**

**

**





**

**

**

**

**Table S1.** **Limit of detection (*LOD)* and limit of quantitation (*LOQ)* of AGEs.**

|  | *LOD* | *LOQ* |
| --- | --- | --- |
|  | pmol/µmol leucine equivalent | pmol/µmol leucine equivalent |
| CML | 49.58 | 148.74 |
| NFL | 9.19 | 27.57 |
| CMA | 10.04 | 30.12 |
| NAL | 12.72 | 38.16 |
| CEA | 5.62 | 16.86 |
| MG-H1 | 31.45 | 94.35 |
| Glucosepane | 1.96 | 5.88 |
| Pyrraline | 0.19 | 0.57 |
| MODIC | 0.01 | 0.03 |

**Table S2. AGE levels^#^ in young vs. aged human lens capsules.**

**Posterior capsule**

|  | **CML** | **NAL** | **NFL** | **Pyrraline** | **MG-H1** | **CEA** | **CMA** | **MODIC** | **Glucosepane** |
| --- | --- | --- | --- | --- | --- | --- | --- | --- | --- |
| Young (<30 years) | 4457.14 ± 810.54 | 1589.73 ± 270.64 | 1049.88 ± 365.83 | 66.38 ± 48.92 | 3696.10 ± 1871.26 | 1544.18 ± 500.09 | 3256.27 ± 592.18 | 4.74 ± 1.28 | 169.79 ± 56.36 |
| Aged (>60 years) | 8915.05 ± 1614.37** | 2457.81 ± 780.44 | 1976.47 ± 566.13* | 64.27 ± 44.08 | 6415.41 ± 2467.71 | 2455.58 ± 1126.58 | 2874.09 ± 1158.59 | 7.77 ± 4.28 | 430.04 ± 88.34** |

* p < 0.05, ** p < 0.005

**Anterior capsule**

|  | **CML** | **NAL** | **NFL** | **Pyrraline** | **MG-H1** | **CEA** | **CMA** | **MODIC** | **Glucosepane** |
| --- | --- | --- | --- | --- | --- | --- | --- | --- | --- |
| Young (<30 years) | 4606.85 ± 462.23 | 1339.55 ± 335.38 | 1169.13 ±226.71 | 29.74 ±3.56 | 2647.05 ± 236.94 | 1224.55 ± 130.26 | 3482.31 ± 465.24 | 5.88 ± 2.80 | 180.78 ± 50.37 |
| Aged (>60 years) | 7613.28 ±2191.79 | 2846.53 ±1180.60 | 1851.25 ±556.82 | 67.12 ±33.03 | 6133.96 ±2116.96* | 2911.02 ±1079.65* | 4130.10 ±1404.42 | 9.71 ±3.48 | 486.86 ± 225.58 |

* p < 0.05

# ^#^pmol/μmol leucine equivalent

# Table S3. List of primers used for qPCR.

| **Gene** | **Forward Primer** | **Reverse Primer** | **Reference** |
| --- | --- | --- | --- |
| hsa-miR-204 | 5’-ACACTCCAGCTGGGTTC CCTTTGTCATCCT- 3’ | 5’-CTCAACTGGTGTCGTG GAGTCGGCAATTCAGTTGAGAGGCATAG -3’ | ([Wang *et al.* 2013](#_ENREF_4)) |
| hsa-miR-184 | 5’-ACACTCCAGCTGGGTGG ACGGAGAACTGAT-3’ | 5’-CTCAACTGGTGTCGTG GAGTCGGCAATTCAGTTGAGACCCTTAT- 3’ | ([Wang *et al.* 2013](#_ENREF_4)) |
| BMP4 | 5’-AGGAAGCAGTCTGTGTA GTGTG -3’ | 5’-GATGGTAGTAGAGGGA TGTGGG- 3’ | ([Dawes *et al.* 2007](#_ENREF_1)) |
| α-SMA | 5’-TTCAATGTCCCAGCCAT GTA-3’ | 5’-GAAGGAATAGCCACGC TCAG- 3’ | ([Wang *et al.* 2013](#_ENREF_4)) |
| hsa-miR-4279 | 5’-ACACTCCAGCTGGGCTC TCCTCCC- 3’ | 5’-CTCAACTGGTGTCGTG GAGTCGGCAATTCAGTTGAG GAAGCCGG- 3’ | ([Wang *et al.* 2013](#_ENREF_4)) |
| hsa-miR-1469 | 5’-ACACTCCAGCTGGGCUC GGCGCGGGGCGCG- 3’ | 5’-CTCAACTGGTGTCGTG GAGTCGGCAATTCAGTTGAG GGAGCCCG- 3’ | ([Wang *et al.* 2013](#_ENREF_4)) |
| GAPDH | 5’-GTCAGTGGTGGACCTGA CCT- 3’ | 5’-TGCTGTAGCCAAATTC GTTG- 3’ | ([Wang *et al.* 2013](#_ENREF_4)) |
| MMP2 | 5’-CACCCATTTACACCTAC ACC- 3’ | 5’-GTTTTTGCTCCAGTTA AAGG- 3’ | ([Dawes *et al.* 2007](#_ENREF_1)) |
| Smad4 | 5’-GACTGAGGTCTTTTACC GTTGG- 3’ | 5’-CTTCAAGCTCTGAGCC ATGC- 3’ | ([Dawes *et al.* 2007](#_ENREF_1)) |
| Smad7 | 5’-AAAGTGTTCCCTGGTTT CTCCATCAAGGC -3’ | 5’-CTACCGGCTGTTGAAG ATGACCTCCAGCCAGCAC- 3’ | ([Dawes *et al.* 2007](#_ENREF_1)) |
| CTGF | 5’-AACTATGATTAGAGCCA ACTGCCTG- 3’ | 5’-TCATGCCATGTCTCCG TACATCTTC- 3’ | ([Dawes *et al.* 2007](#_ENREF_1)) |
| Integrin α5 | 5′-CAGGGTGGTGCTGTCTA CCT-3′ | 5′-GCTCAGTGGCTCCTTCT CTG-3′ | ([Wong *et al.* 2010](#_ENREF_5)) |
| Integrin αV | 5′-ATGGCAAACTCCAAGAG GTG-3′ | 5′-GAGATGGGACTGCGTT CAAG-3′ | ([Wong *et al.* 2010](#_ENREF_5)) |
| Integrin β1 | 5’-CAAGAGAGCTGAAGAC TATCCCA-3’ | 5’-TGAAGTCCGAAGTAAT CCTCCT-3’ | ([Spandidos *et al.* 2010](#_ENREF_2)) |
| TGFβR1 | 5’-GCTGTATTGCAGACTTA GGACTG-3’ | 5’-TTTTTGTTCCCACTCTG TGGTT-3’ | ([Spandidos *et al.* 2010](#_ENREF_2)) |
| TGFβR2 | 5’-AAGATGACCGCTCTGAC ATCA-3’ | 5’-CTTATAGACCTCAGCA AAGCGAC-3’ | ([Spandidos *et al.* 2010](#_ENREF_2)) |
| TGFβR3 | 5′-GCAAAGTGGCATCATAT TATTCC-3’ | 5′-TGCCTTACTTCTCTTGC CTTAA-3’ | ([Dawes *et al.* 2007](#_ENREF_1)) |
| Fibronectin | 5′-CTGGAACCGGGAACCG AATATA-3′ | 5′-TTCTTGTCCTACATTCG GCGG-3′ | ([Spandidos *et al.* 2010](#_ENREF_2)) |

**Table S4. Fold change in the mRNA levels of EMT-associated proteins in HLE cells cultured on unmodified and AGE-modified BME and treated with TGFβ2.**

| **Group** | **Unmodified BME**  **1** | **Unmodified BME + TGF**β**2**  **2** | **AGE-modified BME**  **3** | **AGE-modified BME + TGF**β**2**  **4** |
| --- | --- | --- | --- | --- |
| Smad 4 | 1.00 ± 0.00 | 1.16 ± 0.06** | 1.13 ± 0.13^#^ | 1.34 ± 0.07***^,††^ |
| Smad 7 | 1.00 ± 0.00 | 6.19 ± 0.32*** | 2.85 ± 1.42^#,#^ | 7.29 ± 0.61**^,†^ |
| Integrin α5 | 1.00 ± 0.00 | 1.40 ± 0.32** | 1.04 ± 0.11 | 1.52 ± 0.13** |
| Integrin β1 | 1.00 ± 0.00 | 1.75 ± 0.23*** | 1.07 ± 0.11 | 1.80 ± 0.05** |
| TGFβR1 | 1.00 ± 0.00 | 3.00 ± 0.08*** | 1.12 ± 0.09 | 4.00 ± 0.49***^,†††^ |
| Fibronectin | 1.00 ± 0.00 | 1.26 ± 0.37** | 1.25 ± 0.03^#^ | 1.30 ± 0.11** |

** p< 0.005, *** p< 0.0005-between groups 1 and 2 and groups 3 and 4. ^†^p< 0.05, ^††^p< 0.005, ^†††^p< 0.0005-between groups 2 and 4. ^#^ p< 0.05, ^##^ p< 0.005-between groups 1 and 3.

**Table S5. Fold change in the mRNA levels of EMT-associated proteins in HLE cells cultured on unmodified and AGE-modified lens capsules and treated with TGFβ2**.

| **Groups** | **Unmodified capsule**  **1** | **Unmodified capsule + TGF**β**2**  **2** | **AGE-modified capsule**  **3** | **AGE-modified capsule + TGF**β**2**  **4** |
| --- | --- | --- | --- | --- |
| Smad 4 | 1.00 ± 0.00 | 1.40 ± 0.24** | 1.26 ± 0.21^#^ | 1.96 ± 0.31***^,†††^ |
| Smad 7 | 1.00 ± 0.00 | 4.81 ± 0.12*** | 0.97 ± 0.08 | 5.63 ± 0.39***^,†††^ |
| Integrin α5 | 1.00 ± 0.00 | 1.30 ± 0.09*** | 1.07 ± 0.07 | 1.53 ± 0.13***^,†††^ |
| Integrin β1 | 1.00 ± 0.00 | 1.28 ± 0.09*** | 0.87 ± 0.13^#^ | 1.35 ± 0.09*** |
| TGFβR1 | 1.00 ± 0.00 | 1.96 ± 0.09*** | 0.98 ± 0.05 | 1.99 ± 0.13*** |
| Fibronectin | 1.00 ± 0.00 | 1.86 ± 0.29*** | 1.49 ± 0.05^###^ | 2.28 ± 0.16***^,†††^ |

** p< 0.005, *** p< 0.0005-between groups 1 and 2 and groups 3 and 4. ^†††^p< 0.0005-between groups 2 and 4. ^#^ p< 0.05, ^###^ p< 0.0005-between groups 1 and 3.

**Table S6. Individual and total AGE levels^#^ in capsule specimens from capsular bags**

| **Donor age (years)** | **CML** | **NAL** | **NFL** | **Pyrraline** | **MG-H1** | **CEA** | **CMA** | **MODIC** | **Total AGEs** |
| --- | --- | --- | --- | --- | --- | --- | --- | --- | --- |
| 15 | 7162.82 | 2490.02 | 1598.03 | 48.91 | 4827.27 | 1923.00 | 2851.37 | 13.50 | 20914.92 |
| 43 | 18503.28 | 2229.49 | 2164.18 | 98.94 | 4944.69 | 3019.37 | 1674.88 | 14.16 | 32648.99 |
| 44 | 10281.58 | 1602.14 | 1969.36 | 91.63 | 3535.64 | 5830.53 | 1524.11 | 13.61 | 24848.60 |
| 57 | 10977.59 | 2692.26 | 1260.85 | 86.92 | 2969.13 | 2517.03 | 975.79 | 13.11 | 21492.68 |
| 64 | 11827.22 | 2971.03 | 2115.16 | 73.19 | 3380.60 | 1377.27 | 1313.08 | 15.42 | 23072.97 |
| 77 | 20871.02 | 9410.95 | 6787.51 | 126.71 | 18287.89 | 7432.86 | 10164.68 | 45.49 | 73127.12 |
| 78 | 12010.03 | 2467.57 | 2500.53 | 61.57 | 5687.20 | 2255.89 | 2451.56 | 15.80 | 27450.14 |

^#^pmol/μmol leucine equivalent

**References**

Dawes LJ, Elliott RM, Reddan JR, Wormstone YM, Wormstone IM (2007). Oligonucleotide microarray analysis of human lens epithelial cells: TGFbeta regulated gene expression. *Mol. Vis.* **13**, 1181-1197.

Spandidos A, Wang X, Wang H, Seed B (2010). PrimerBank: a resource of human and mouse PCR primer pairs for gene expression detection and quantification. *Nucleic Acids Res.* **38**, D792-799.

Thornalley PJ, Rabbani N (2014). Detection of oxidized and glycated proteins in clinical samples using mass spectrometry--a user's perspective. *Biochim. Biophys. Acta*. **1840**, 818-829.

Wang Y, Li W, Zang X, Chen N, Liu T, Tsonis PA, Huang Y (2013). MicroRNA-204-5p regulates epithelial-to-mesenchymal transition during human posterior capsule opacification by targeting SMAD4. *Invest. Ophthalmol. Vis. Sci.* **54**, 323-332.

Wong JC, Gao SY, Lees JG, Best MB, Wang R, Tuch BE (2010). Definitive endoderm derived from human embryonic stem cells highly express the integrin receptors alphaV and beta5. *Cell adhesion & migration*. **4**, 39-45.
